# Supplementary material for: Dissection and function of autoimmunity-associated TNFAIP3 (A20) gene enhancers in humanized mouse models
Source: Nat Commun. 2018 Feb 13;9:658. doi: 10.1038/s41467-018-03081-7 (PMC5811492; doi:10.1038/s41467-018-03081-7)

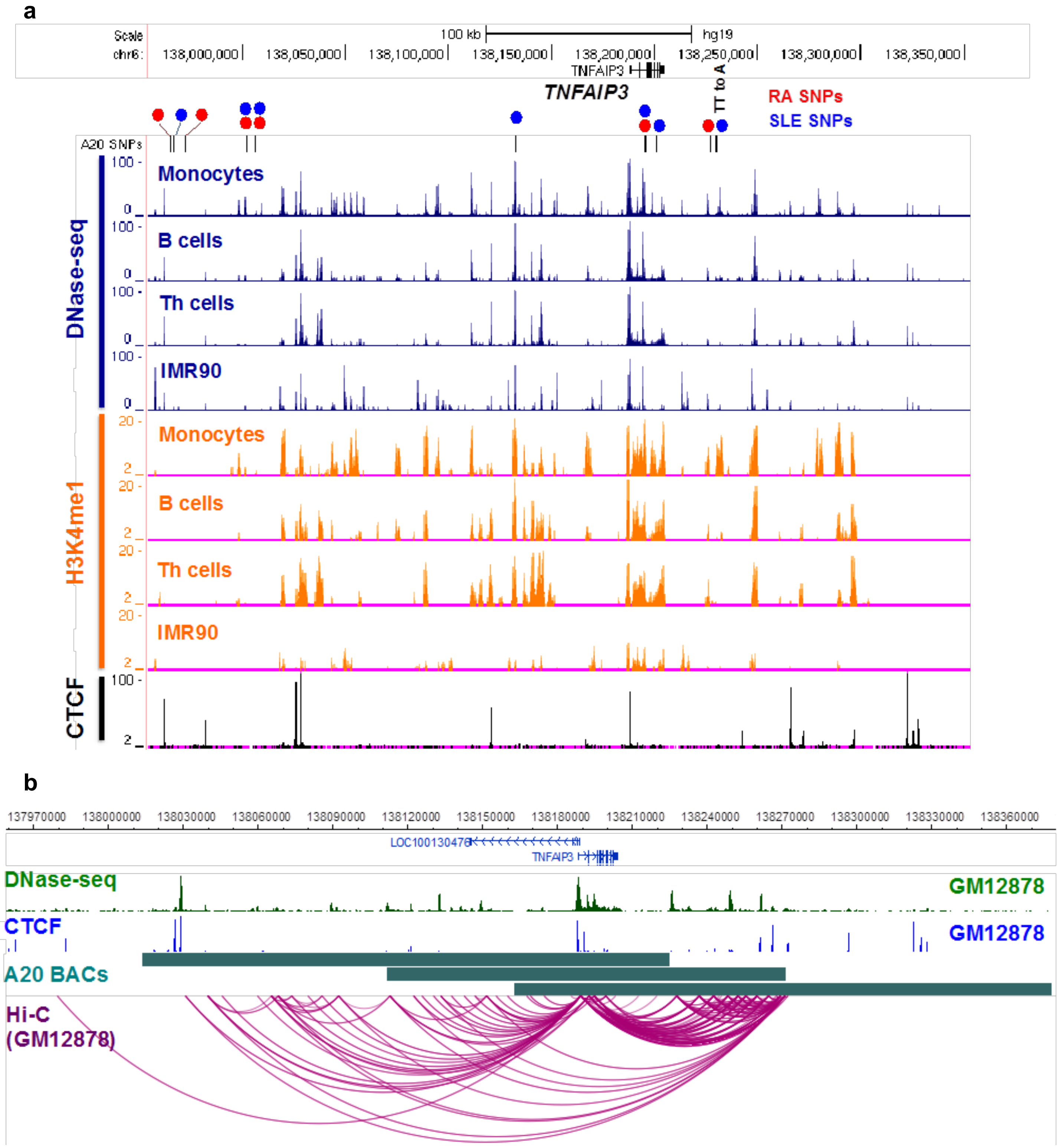

Supplementary Figure 1.

Epigenomic analysis of human *TNFAIP3/A20* locus. (**a**) Gene tracks of chromatin accessibility (DNase-seq) or read counts of H3K4me1 or CTCF from ChIP-seq experiments. (**b**) Visualization of looping interactions based on Hi-C data. Data derived from ENCODE, NIH Roadmap and ref. 23.

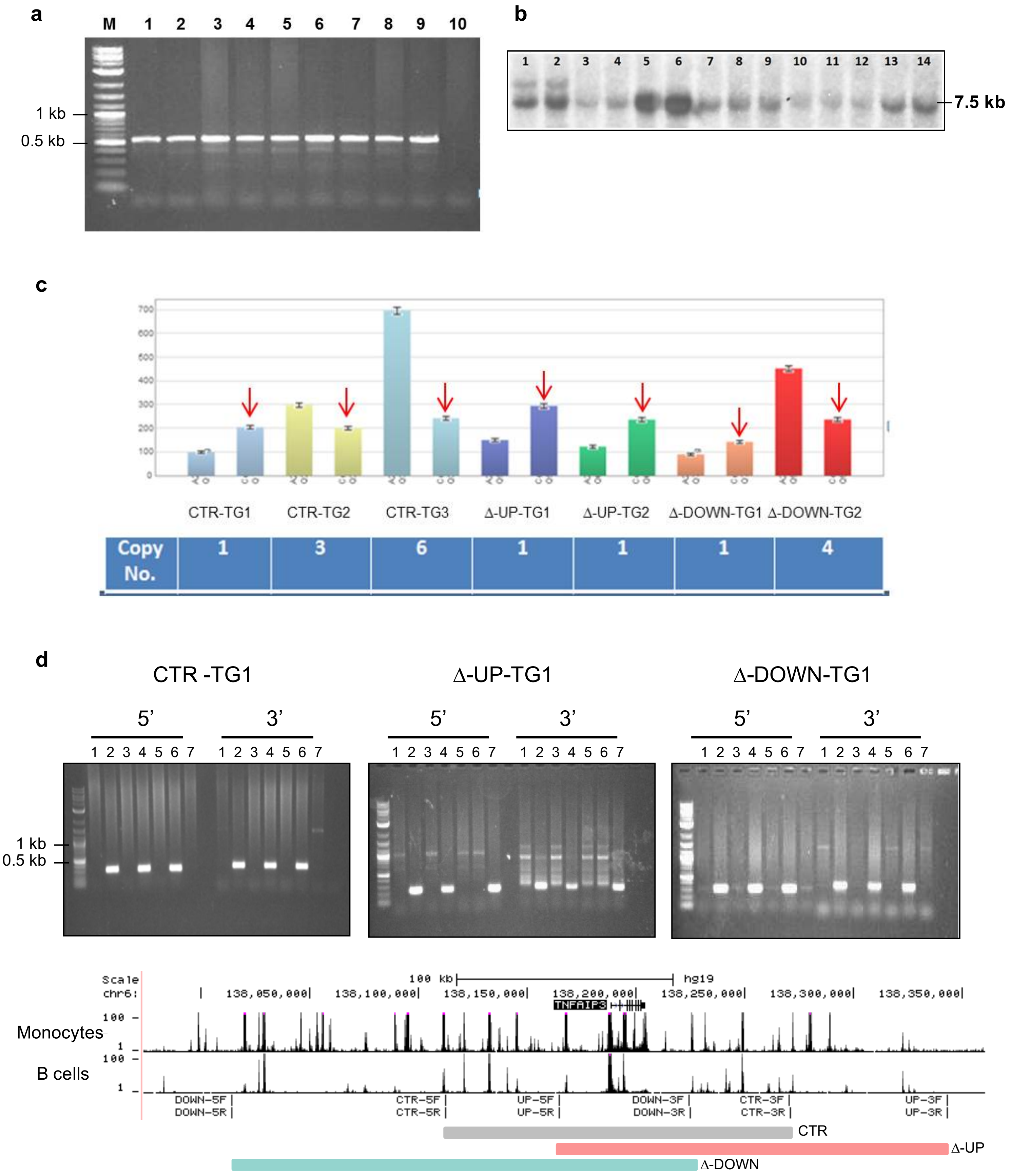
a
M 1 2 3 4 5 6 7 8 9 10
1 kb
0.5 kb
b
1 2 3 4 5 6 7 8 9 10 11 12 13 14
7.5 kb
c
700
600
500
400
300
200
100
0
CTR-TG1 CTR-TG2 CTR-TG3 Δ-UP-TG1 Δ-UP-TG2 Δ-DOWN-TG1 Δ-DOWN-TG2
Copy No. 1 3 6 1 1 1 4
d
CTR -TG1
Δ-UP-TG1
Δ-DOWN-TG1
5' 3'
1 2 3 4 5 6 7 1 2 3 4 5 6 7
1 kb
0.5 kb
Scale 100 kb hg19
chr6: 138,050,000 138,100,000 138,150,000 138,200,000 138,250,000 138,300,000 138,350,000
TNFAIP3
Monocytes
B cells
100
1
DOWN-5F DOWN-5R
CTR-5F CTR-5R
UP-5F UP-5R
DOWN-3F DOWN-3R
CTR-3F CTR-3R
UP-3F UP-3R
CTR
Δ-UP
Δ-DOWN

Supplementary Figure 2.

Characterization of transgenic mice containing the human A20 locus. (**a**) PCR analysis of transgenic founders using human specific A20 primers. M: 2 Log DNA ladder. 1. CTR-TG1; 2. CTR-TG2; 3. CTR-TG3; 4. Δ-UP-TG1; 5. Δ-UP-TG2; 6. Δ-UP-TG3; 7. Δ-DOWN-TG1; 8. Δ-DOWN-TG2; 9. Δ-DOWN-TG3 10. Negative Control. (**b**) Southern blot analysis of PCR positive F1 mice as described in (**a**). DNA was digested with EcoRI, separated by electrophoresis on a 0.7% agarose gel, and transferred to nylon membranes. The blots were analyzed with a probe amplified from human A20 BAC clone containing a sequence immediately upstream of the ATG start codon. 1,2 - CTR-TG1; 3,4 - CTR-TG2; 5,6 - CTR-TG3; 7,8 - Δ-UP-TG1; 9,10 - Δ-UP-TG2; 11,12 - Δ-DOWN-TG1. 13, 14 - Δ-DOWN-TG2. (**c**) Transgene copy number determination by Taqman digital PCR. Genomic DNA from transgenic lines was analyzed by quantitative Taqman digital PCR assay to determine the transgene copy number. Both the target assay and the reference assay are in the same reaction. Transferrin receptor gene is used as control which has two copies in each line (marked by red arrow). The transgene copy number determined for each transgenic line is indicated. The results shown in this study were obtained from mice with one copy of the transgene, labeled 'TG1' in this figure. (**d**) PCR screen for the integrity of the BAC transgene. The odd-numbered lanes are for negative controls, the even-numbered lanes are for BAC transgenic mice. The PCR primers used for each region and size of product are as follows: for CTR-TG1: 5 prime F and R (299 bp); 3 prime F and R (402 bp); for Δ-UP-BAC-TG1: 5 primer F and R (211 bp); 3 primer F and R (257 bp); for Δ-DOWN-BAC-TG1: 5 primer F and R (302 bp); 3 primer F and R (358 bp). The panel below shows the genomic locations of the primers used relative to the position of the 3 BACs. DNase-seq tracks are derived from ENCODE.

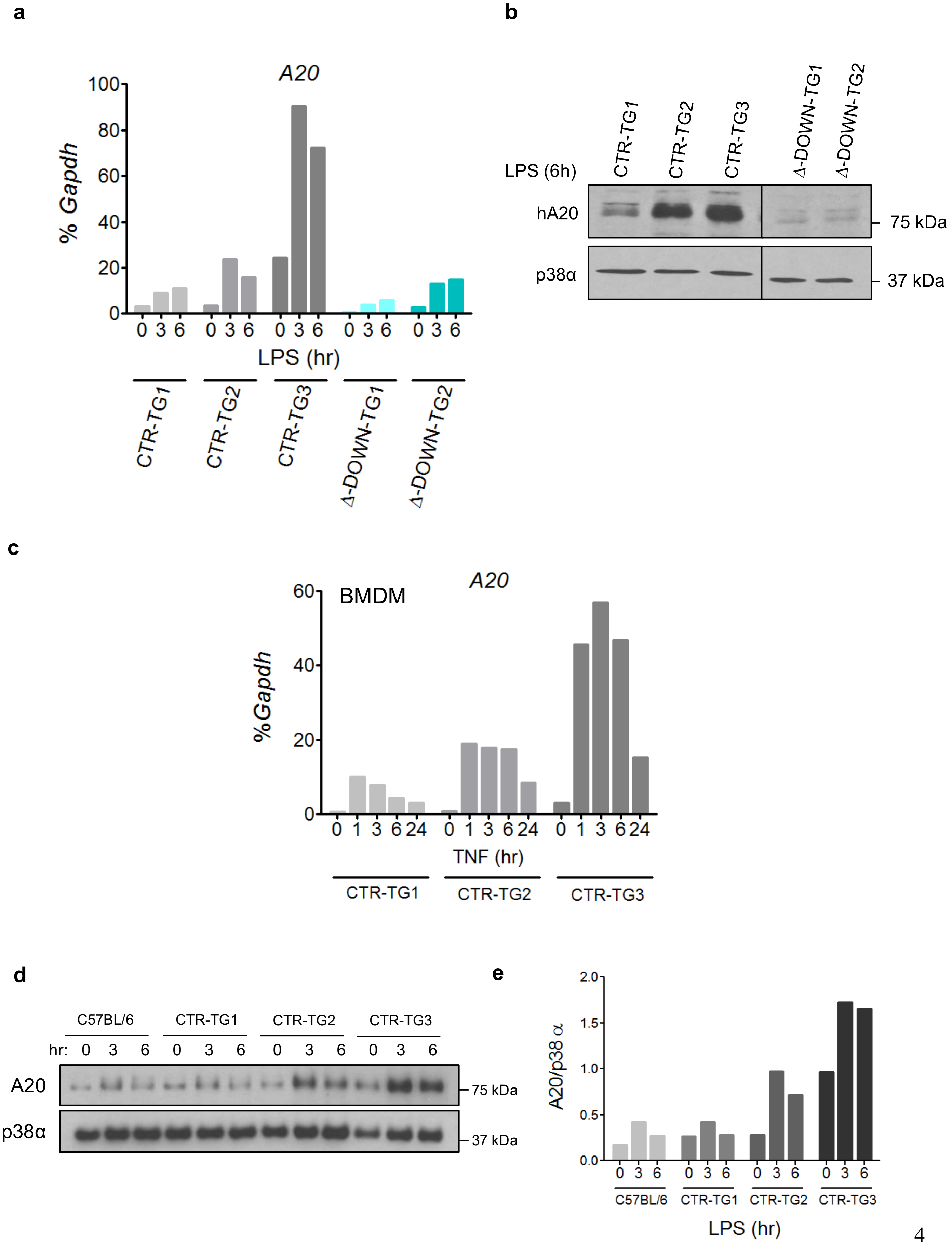
a
A20
% Gapdh
100
80
60
40
20
0
0 3 6 0 3 6 0 3 6 0 3 6 0 3 6
LPS (hr)
CTR-TG1
CTR-TG2
CTR-TG3
Δ-DOWN-TG1
Δ-DOWN-TG2
b
LPS (6h)
CTR-TG1
CTR-TG2
CTR-TG3
Δ-DOWN-TG1
Δ-DOWN-TG2
hA20
p38α
75 kDa
37 kDa
c
BMDM
A20
%Gapdh
60
40
20
0
0 1 3 6 24 0 1 3 6 24 0 1 3 6 24
TNF (hr)
CTR-TG1
CTR-TG2
CTR-TG3
d
C57BL/6
CTR-TG1
CTR-TG2
CTR-TG3
hr: 0 3 6 0 3 6 0 3 6 0 3 6
A20
p38α
75 kDa
37 kDa
e
A20/p38 α
2.0
1.5
1.0
0.5
0.0
0 3 6 0 3 6 0 3 6 0 3 6
C57BL/6
CTR-TG1
CTR-TG2
CTR-TG3
LPS (hr)

Supplementary Figure 3.
Copy number-dependent hA20 expression in transgenic mouse lines.
(**a**) mRNA expression of *hA20* in BMDM derived from CTR-TG1 (one copy), CTR-TG2 (three copies), CTR-TG3(6 copies), Δ-DOWN-TG1 (one copy) and TG2 (4 copies) mouse lines at the indicated time points with or without stimulation with LPS. (**b**) Immunoblot analysis of hA20 expression in whole-cell lysates obtained from BMDM derived from mice as described in (**a**). The vertical black line indicates position of splicing of images from the same gel. (**c**) mRNA expression of *hA20* in BMDM derived from CTR-TG1 (one copy), CTR-TG2 (three copies) and CTR-TG3(6 copies) mouse lines at the indicated time points with or without stimulation with TNF. (**d**) Immunoblot analysis of hA20 expression in whole-cell lysates obtained from BMDM derived from C57BL/6, CTR-TG1 (one copy), CTR-TG2 (three copies) and CTR-TG3 (6 copies) mouse lines at the indicated time points stimulated with LPS. (**e**) Quantification of A20 protein levels of blot shown in (**d**).

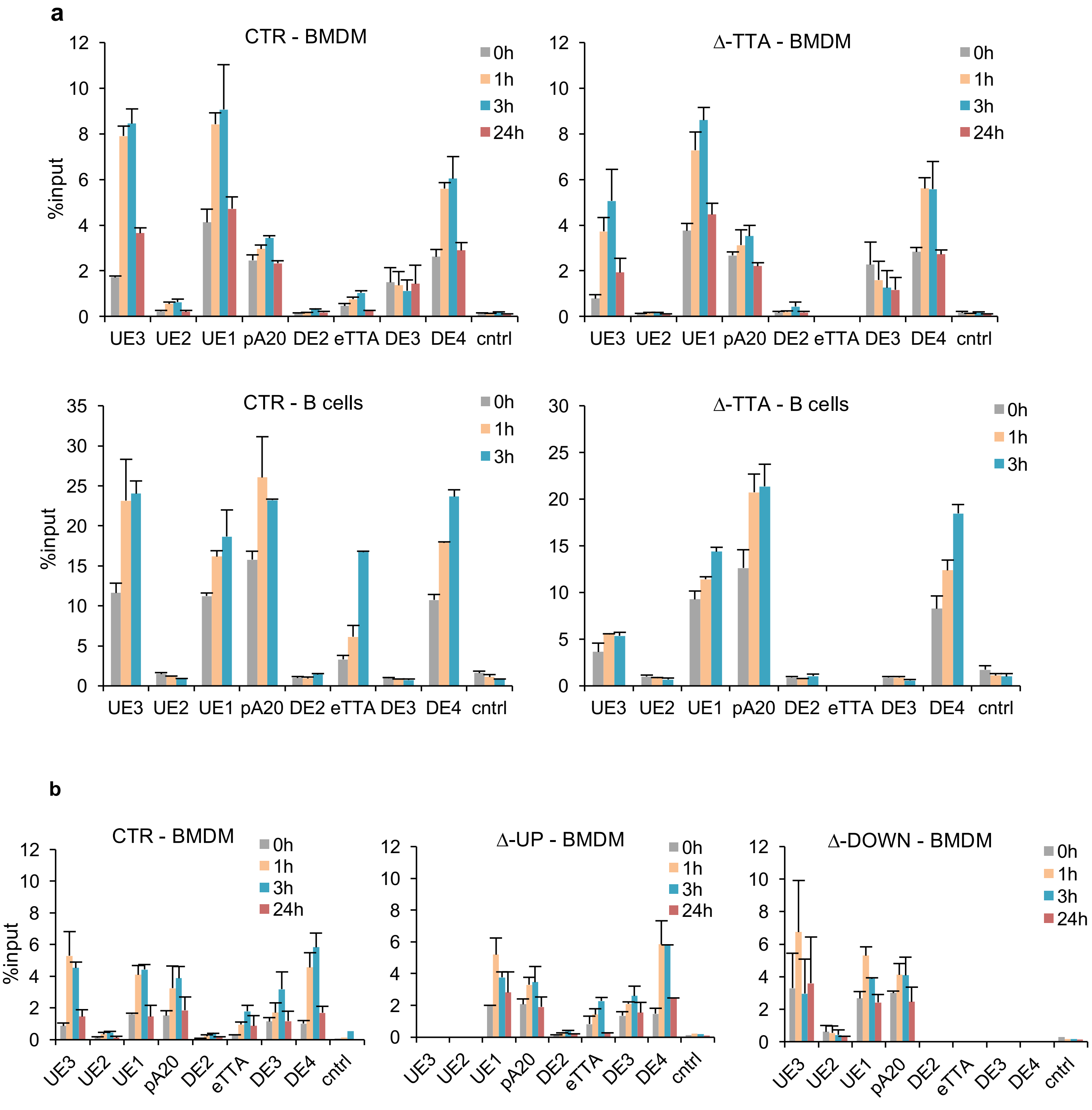

Supplementary Figure 4.

Analysis of chromatin accessibility at the *TNFAIP3* genomic locus in BAC transgenic mice. Evaluation of chromatin accessibility at *TNFAIP3* enhancers and promoter by FAIRE in bone marrow derived macrophages (BMDM) and B-cells incubated with or without LPS (25 ng mL^{-1}) or CD40+IgM respectively, for the times indicated. In (**a**), cells from CTR and Δ-TT>A mice were analyzed in parallel; in (**b**) cells from CTR, Δ-UP and Δ-DOWN mice were analyzed in parallel. Chromatin accessibility is displayed relative to total input. Values represent mean ± SEM. of three (**a**) (BMDM) or two (**a**, B cells and **b**, BMDM) independent experiments. Error bars represent mean ± s.e.m.

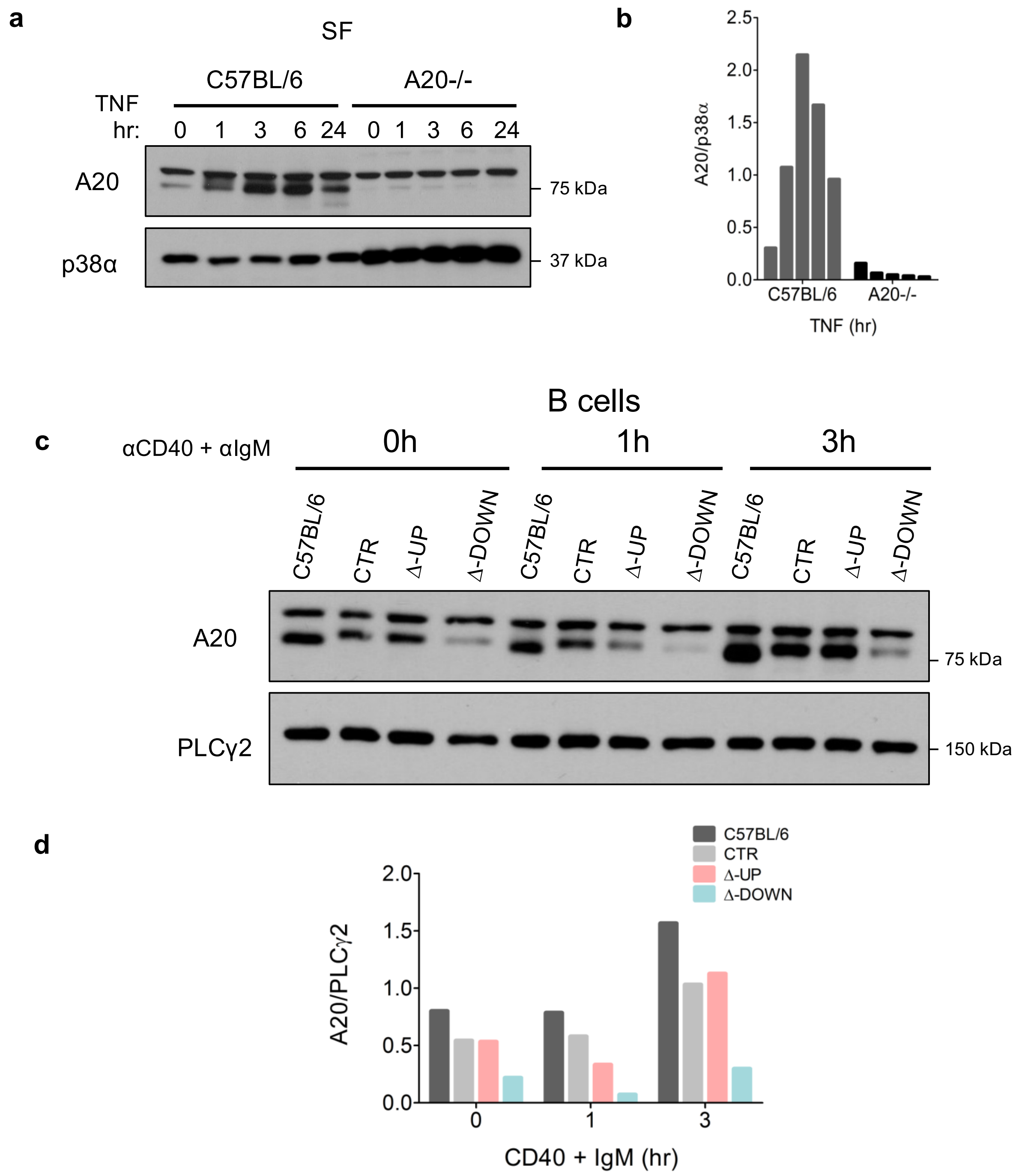

Supplementary Figure 5.

Immunoblot analysis of A20 in transgenic mice.(**a**) Immunoblot analysis of A20 expression in whole-cell lysates obtained from C57BL/6 and A20-/- SFs at the indicated time points after stimulation with mTNF. p38α was used as loading control. Cells were pooled from 2 mice per genotype in each experiment. (**a**) demonstrates that the lower band that corresponds to mA20 is absent in *Tnfaip3*-/- cells. (**b**) Quantitation of A20 protein levels normalized to p38α. (**c**) Decreased expression of hA20 protein in hA20(Δ-DOWN) B cells. Immunoblot analysis of A20 expression in whole-cell lysates obtained from C57BL/6, hA20(CTR), hA20(Δ-UP) and hA20(Δ-DOWN) B-cells at the indicated time points after stimulation with αCD40 and αIgM. PLCγ2 was used as loading control. In (**c**) the transgenic mice were on a mA20-deficient background. (**d**) Quantification of A20 protein levels normalized to PLCγ2.

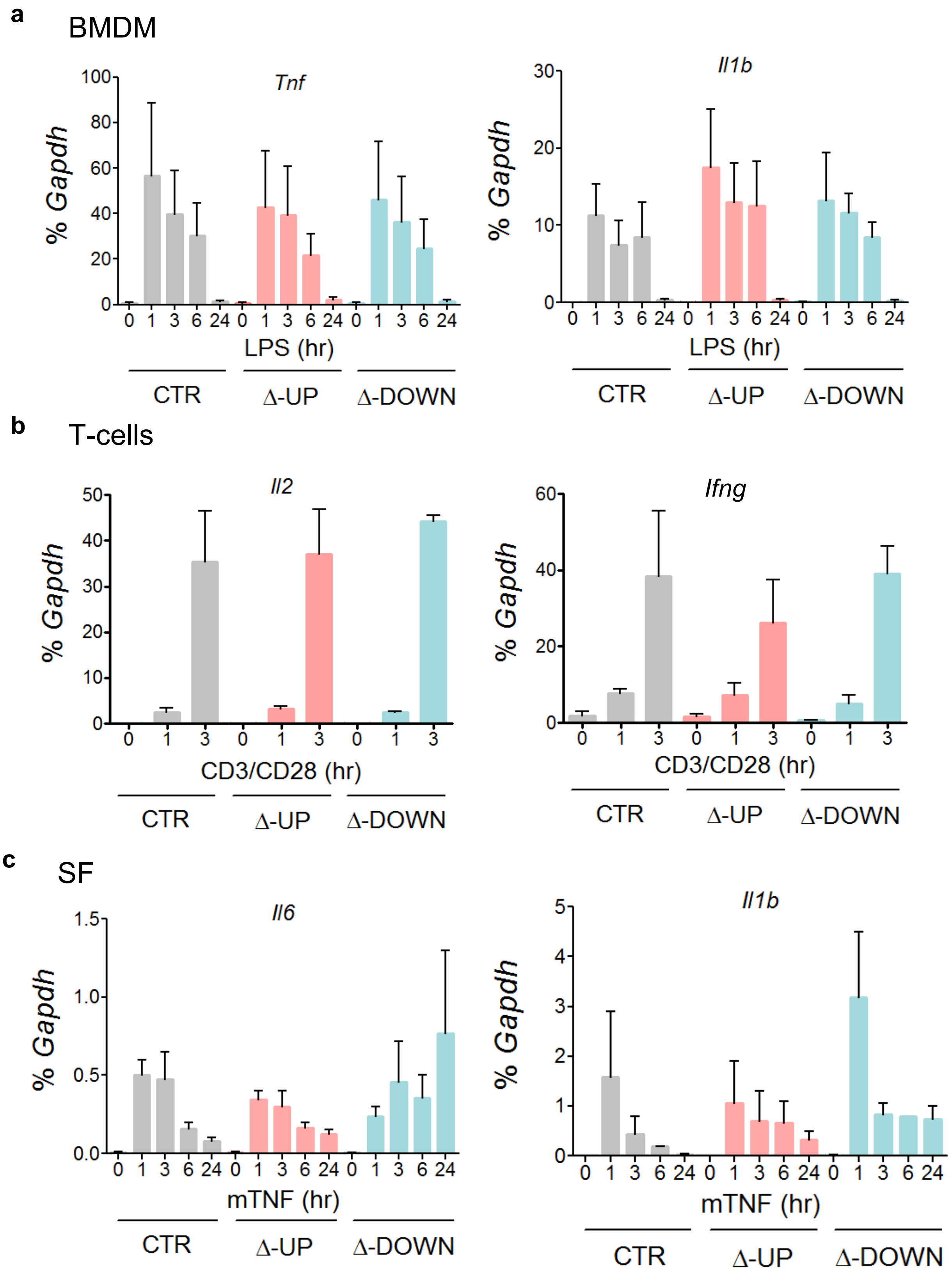

Supplementary Figure 6.

Quantitative RT-PCR analysis of mRNA expression of inflammatory cytokines in (**a**) BMDM, (**b**) T-cells, and (**c**) SFs in the indicated mice. Data are shown as mean ± s.e.m of 2 independent experiments normalized to *mGapdh*, with cells pooled from 2 mice per group in each experiment.

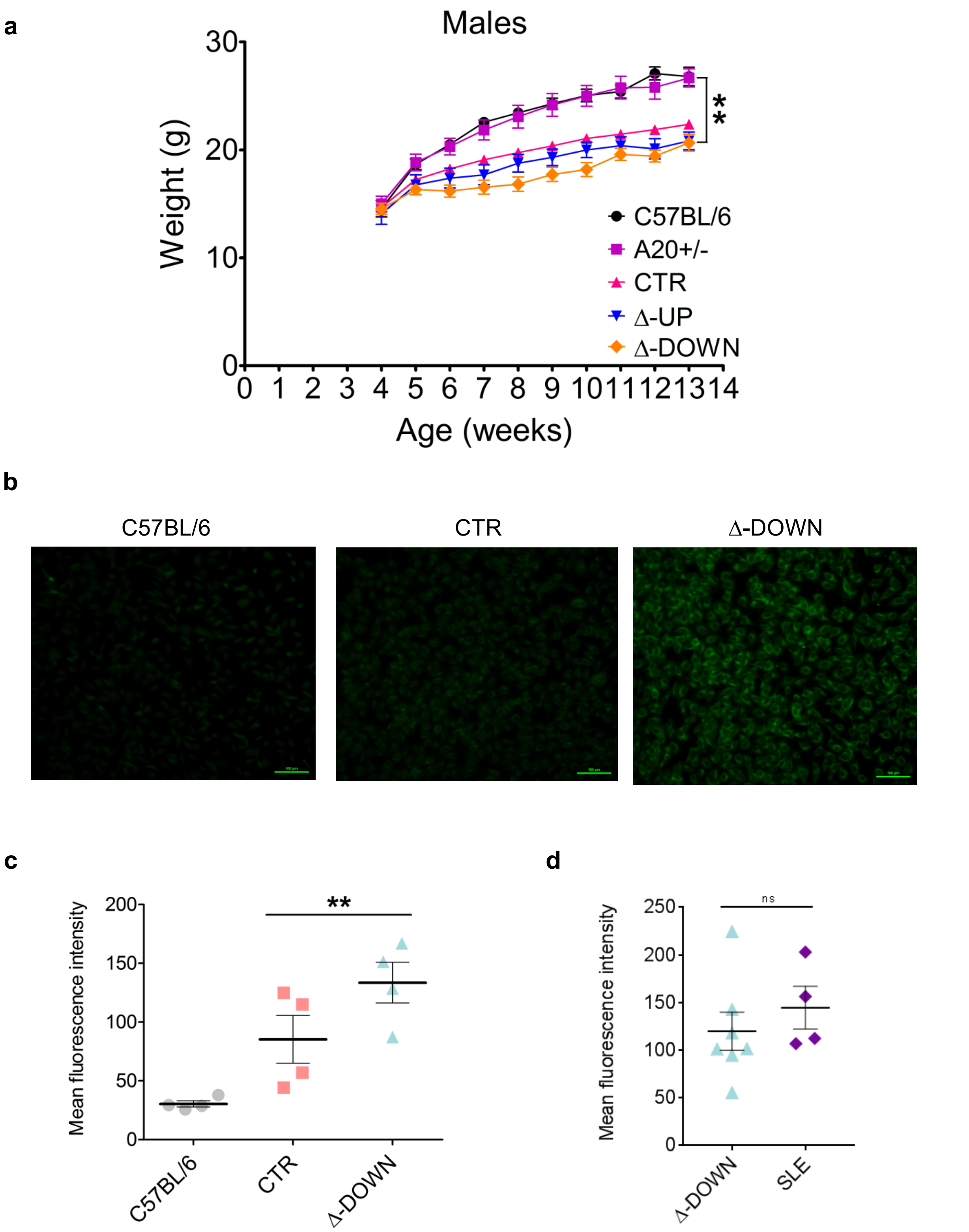

Supplementary Figure 7.

Antinuclear antibody (ANA) development in hA20(Δ-DOWN mice). **(a)** Growth curves of BAC transgenic and control mice. n ≥ 10/Tg group. (**b**) Immunofluorescence for anti-nuclear antibodies (ANAs) in serum of 6-7 month old mice. Representative data from 4 experiments. Image magnification 20X. (**c**) Mean fluorescence intensity of ANAs pooled from 4 independent experiments. ** P< 0.01 (using paired t-test). (**d**) An independent ANA measurement comparing Δ-DOWN (n=7) mice to mice with SLE-like disease (n=4). Error bars represent mean ± s.e.m, unpaired t-test, ns not significant.

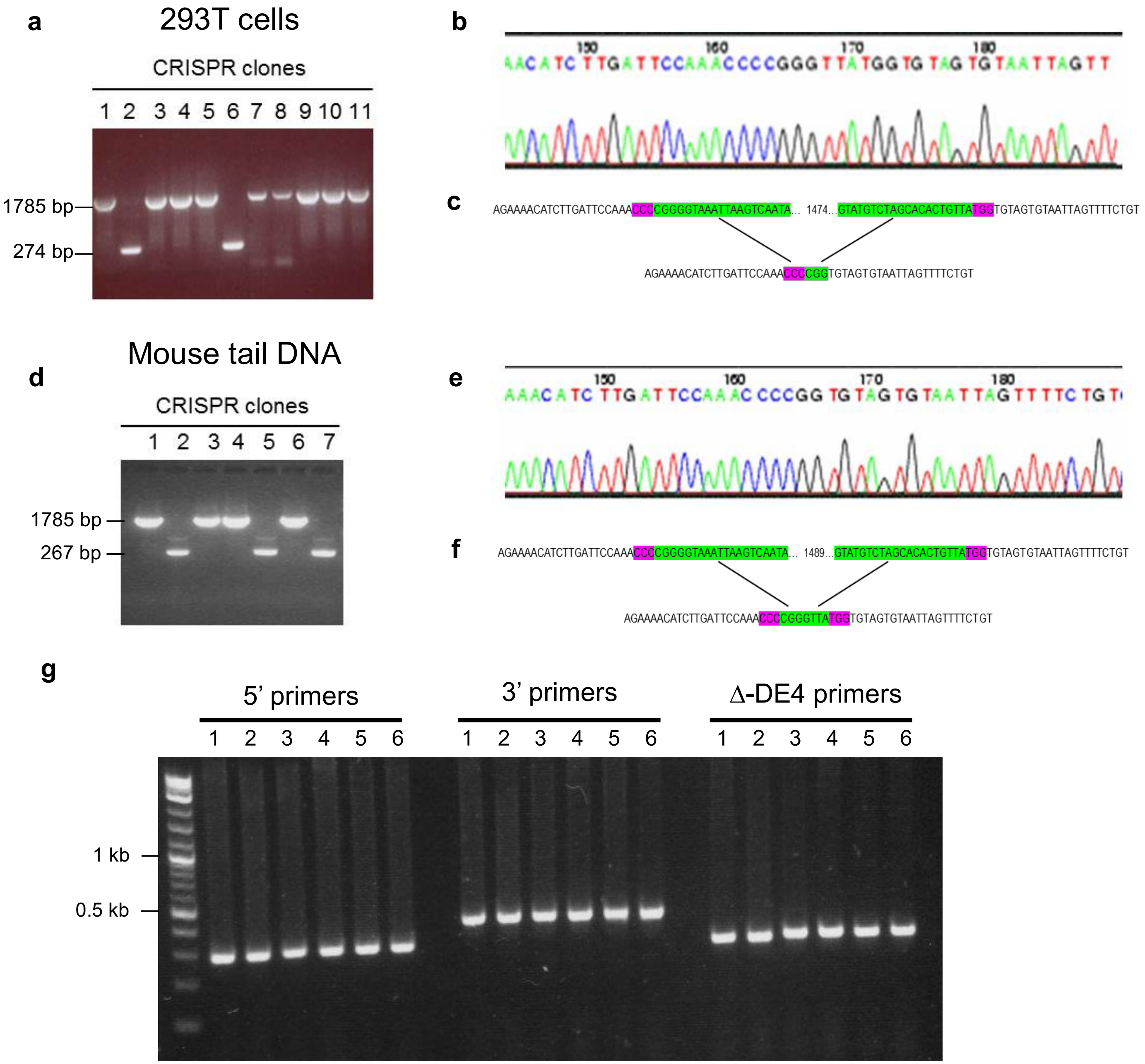

Supplementary Figure 8.

Identification of the DE4 deletion in 293T cells and mouse founders by PCR and Sanger sequencing. (**a**) Identification of DE4 deletion by PCR in 293T cell clones with DE4 primers F and R (wild type,1785 bp; Δ-DE4, ~274 bp). (**b**) Sanger sequencing chromatograms for DE4 deletion in 293T cells. (**c**) Sequences of the wild type and deleted junction in 293T cells as confirmed by Sanger sequencing. The PAM sequences are highlighted in purple. The gRNA sequences are highlighted in green. (**d**) Determination of the DE4 deletion in mouse founders by PCR using human specific A20 primers flanking the deleted region to amplify tail DNA, DE4 primers F and R (wild type,1785 bp; Δ-DE4, ~267 bp). (**e**) Sanger sequencing chromatograms for DE4 deletion in mouse. (**f**) Sequences of the wild type BAC and deleted junction in mouse as confirmed by Sanger sequencing. (**g**) PCR characterization of the integrity of the BAC transgene in Δ-DE4 mice. The PCR primers used for each region and size of product are as follows: 5 prime F and R (299 bp); 3 prime F and R (402 bp). DE4 F and R (~267 bp).

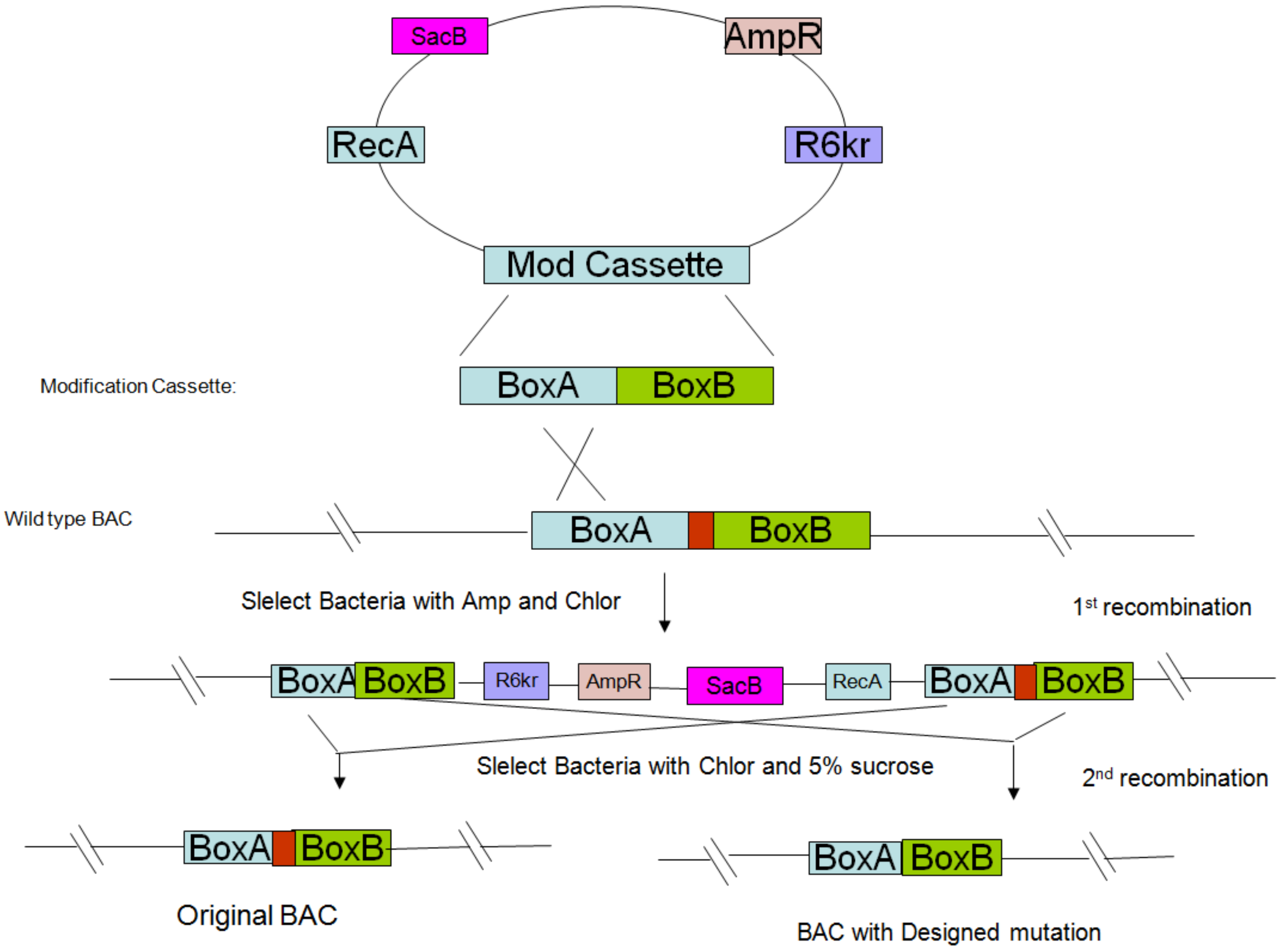

Supplementary Figure 9.

Schematic of two-step BAC modification for TT>A deletion. A and B homology arms were amplified from human A20 CTR-BAC DNA and cloned into BAC shuttle vector, PLD53-SC-AB with the deletion of TT>A enhancer in the middle. The homology arms were used for recombination between the targeting vector PLD53-SC-AB and the wild type BAC. The first recombination is to obtain the co-integrants. The second recombination is to generate either wild type BAC (original) or modified BAC with the correct deletion.

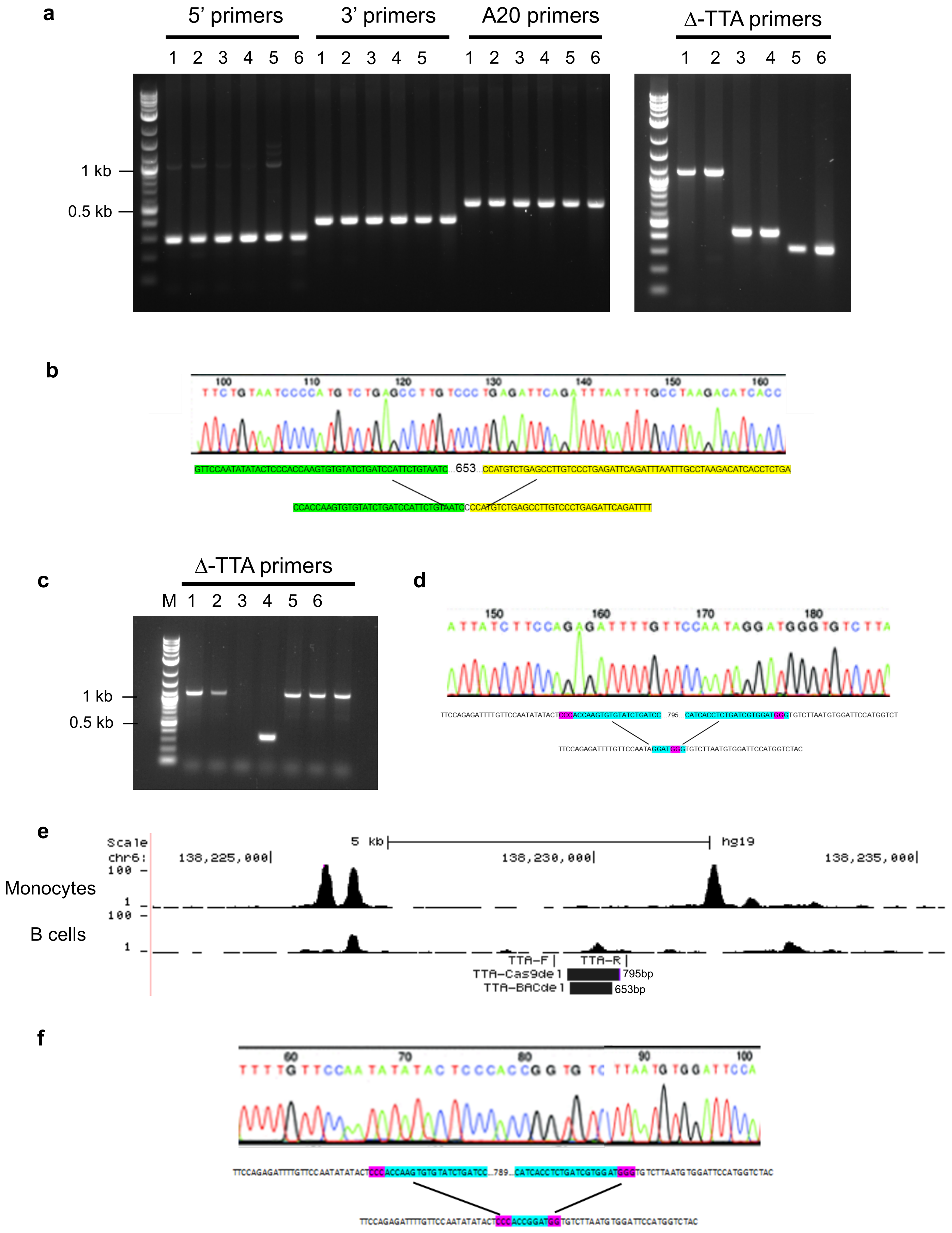
a
5' primers
3' primers
A20 primers
Δ-TTA primers
1 2 3 4 5 6 1 2 3 4 5 1 2 3 4 5 6
1 2 3 4 5 6
1 kb
0.5 kb
b
653
c
Δ-TTA primers
M 1 2 3 4 5 6
1 kb
0.5 kb
d
795
e
Scale
chr6:
138,225,000
5 kb
138,230,000
hg19
138,235,000
Monocytes
B cells
100
1
TTA-F
TTA-R
TTA-Cas9del
795bp
TTA-BACdel
653bp
f
789

Supplementary Figure 10.

Characterization of TT>A enhancer deletions obtained using either BAC modification or CRISPR/Cas9. (**a**) PCR screen for the integrity of the transgene in Δ-TT>A mice. The PCR primers used for each region and size of product are as follows: 5 prime F and R (299 bp); 3 prime F and R (402 bp).A20 primers (524 bp); TT>A F and R (Wild type 1130bp, BAC-ΔTT>A, 477 bp, Cas9-ΔTT>A, 337 bp). 1,2 - CTR-TG1; 3,4 - Δ-TT>A-BAC; 5,6 - Δ-TT>A-Cas9. (**b**) Sanger sequencing chromatograms for the TT>A deletion in BAC-ΔTTA transgenic mouse. Part of A homology arm is highlighted in green and B homology arm is highlighted in yellow (**c**) Dual sgRNA-directed deletion of *TNFAIP3* enhancer harboring TT>A in transgenic mice generated using CRISPR/Cas9. Determination of the deletion in mouse founders by PCR. (**d**) Sanger sequencing chromatograms for the TT>A enhancer deletion in transgenic mouse line (top). Sequences at the sgRNA-targeted site of the wild type CTR-BAC and deleted junction (bottom). PAM sites are underlined and highlighted in red; target sequences are in blue. (**e**) Genomic location relative to DNase-seq tracks obtained from ENCODE of the PCR primers used to detect the TT>A deletion. Horizontal bars beneath the gene tracks depict the regions deleted in the CRISPR-ΔTT>A and BAC-ΔTT>A mouse lines. (**f**) Sanger sequencing chromatograms for the TT>A enhancer deletion in 293T cells (top). Sequences of the wild type and deleted junction in 293T cells as confirmed by Sanger sequencing (bottom).

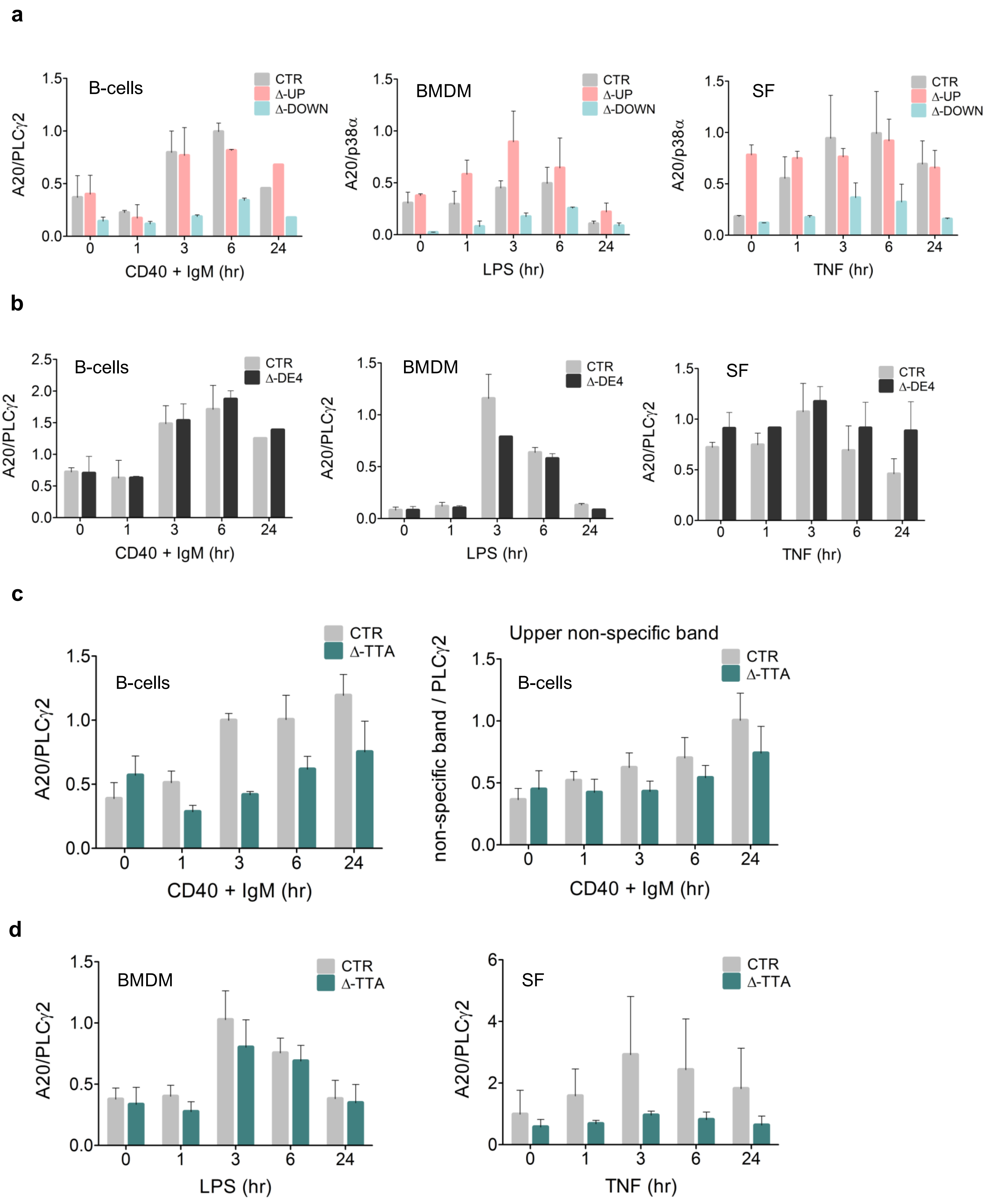
a
B-cells
CTR
Δ-UP
Δ-DOWN
A20/PLCγ2
CD40 + IgM (hr)
BMDM
A20/p38α
LPS (hr)
SF
TNF (hr)
b
B-cells
CTR
Δ-DE4
A20/PLCγ2
CD40 + IgM (hr)
BMDM
LPS (hr)
SF
TNF (hr)
c
CTR
Δ-TTA
B-cells
A20/PLCγ2
CD40 + IgM (hr)
Upper non-specific band
non-specific band / PLCγ2
d
BMDM
LPS (hr)
SF
TNF (hr)

Supplementary Figure 11.

(**a**) Quantitation of A20 protein levels normalized to PLCγ2 or p38α in B-cells, BMDM and SF isolated from CTR, Δ-UP and Δ-DOWN mice at the specified time points. Data are representative of 2 independent experiments, with cells pooled from 2 mice per genotype in each experiment. (**b**) Quantitation of A20 protein levels normalized to PLCγ2 in B-cells, BMDM and SF isolated from CTR and Δ-DE4 mice at the specified time points. Data are representative of 2 independent experiments, with cells pooled from 2 mice per genotype in each experiment. (**c**) Quantitation of A20 protein levels normalized to PLCγ2 in B-cells isolated from CTR and Δ-TT>A mice at the specified time points. Right graph represents quantitation of the upper non-specific band as seen in Figure 5c. Data are pooled from 4 independent experiments, with cells pooled from 2 mice per genotype in each experiment. (**d**) Quantitation of A20 protein levels normalized to PLCγ2 in BMDM and SF isolated from CTR and Δ-TT>A mice at the specified time points. Data are representative of 3 (BMDM) or 2 (SF) independent experiments, with cells pooled from 2 mice per genotype in each experiment. Error bars represent mean ± s.e.m.

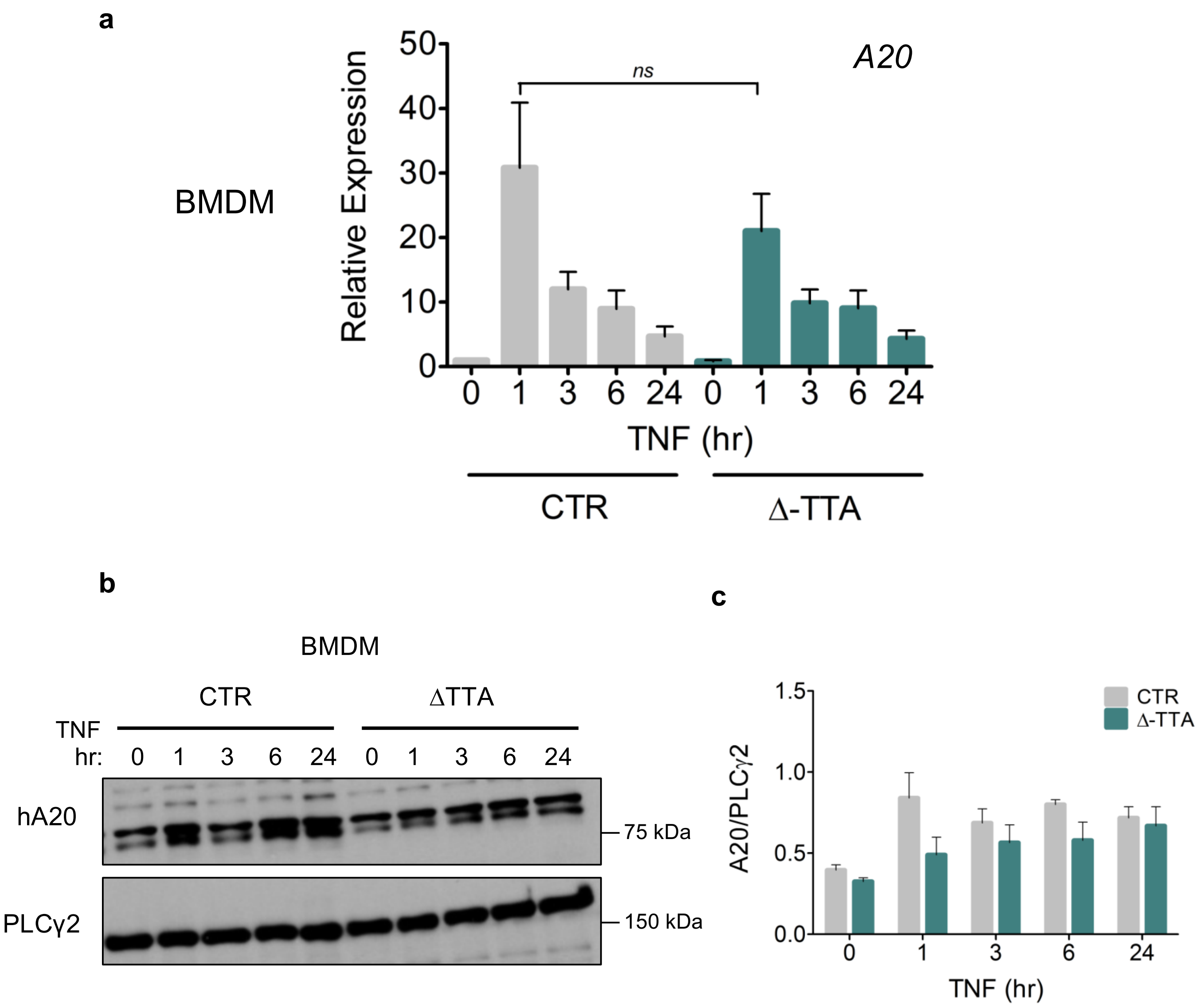

Supplementary Figure 12.

hA20 expression in TNF-stimulated BMDM from hA20(Δ-TT>A) mice. (**a**) mRNA expression of *hA20* in BMDM at the indicated time points after TNF stimulation. Data are shown as mean ± SEM of 3 independent experiments. (**b**) Immunoblot analysis of hA20 expression in whole-cell lysates obtained from CTR and Δ-TT>A BMDMs at the indicated time points after stimulation with TNFα. PLCγ2 was used as loading control. (**c**) Quantitation of A20 protein levels normalized to PLCγ2. Data are representative of 3 independent experiments, with cells pooled from 2 mice per genotype in each experiment. Error bars represent mean ± s.e.m.

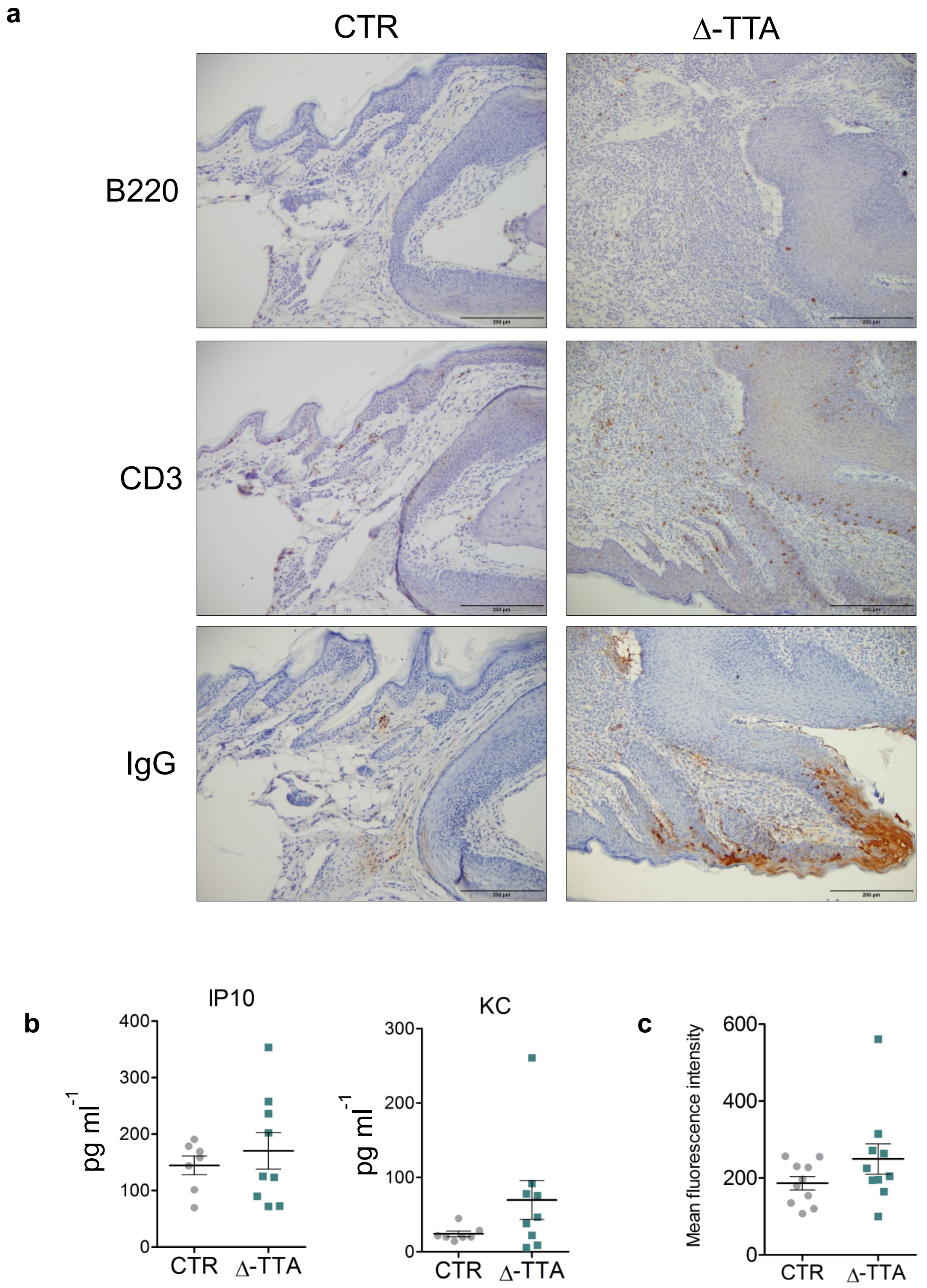

Supplementary Figure 13.

(**a**) Immunohistochemistry analysis of sections of paws and digits from the indicated transgenic mice stained with anti-B220 (B cells), anti-CD3 (T cells) and anti-IgG antibodies showing increased infiltration of T cells and IgG deposition; Scale bars, 200 μm. Photomicrographs are representative of 5 (CTR) and 6 (Δ-TT>A) mice. (**b**) Luminex analysis of CXCL10 and KC protein in serum of aged CTR and Δ-TT>A mice. (**c**) Mean fluorescence intensity of anti-nuclear antibody (ANA) staining using serum of ≥11 month old mice, n = 10. In (**b**) and (**c**) symbols represent individual mice; differences between genotypes were not statistically significant. Error bars represent mean ± s.e.m.

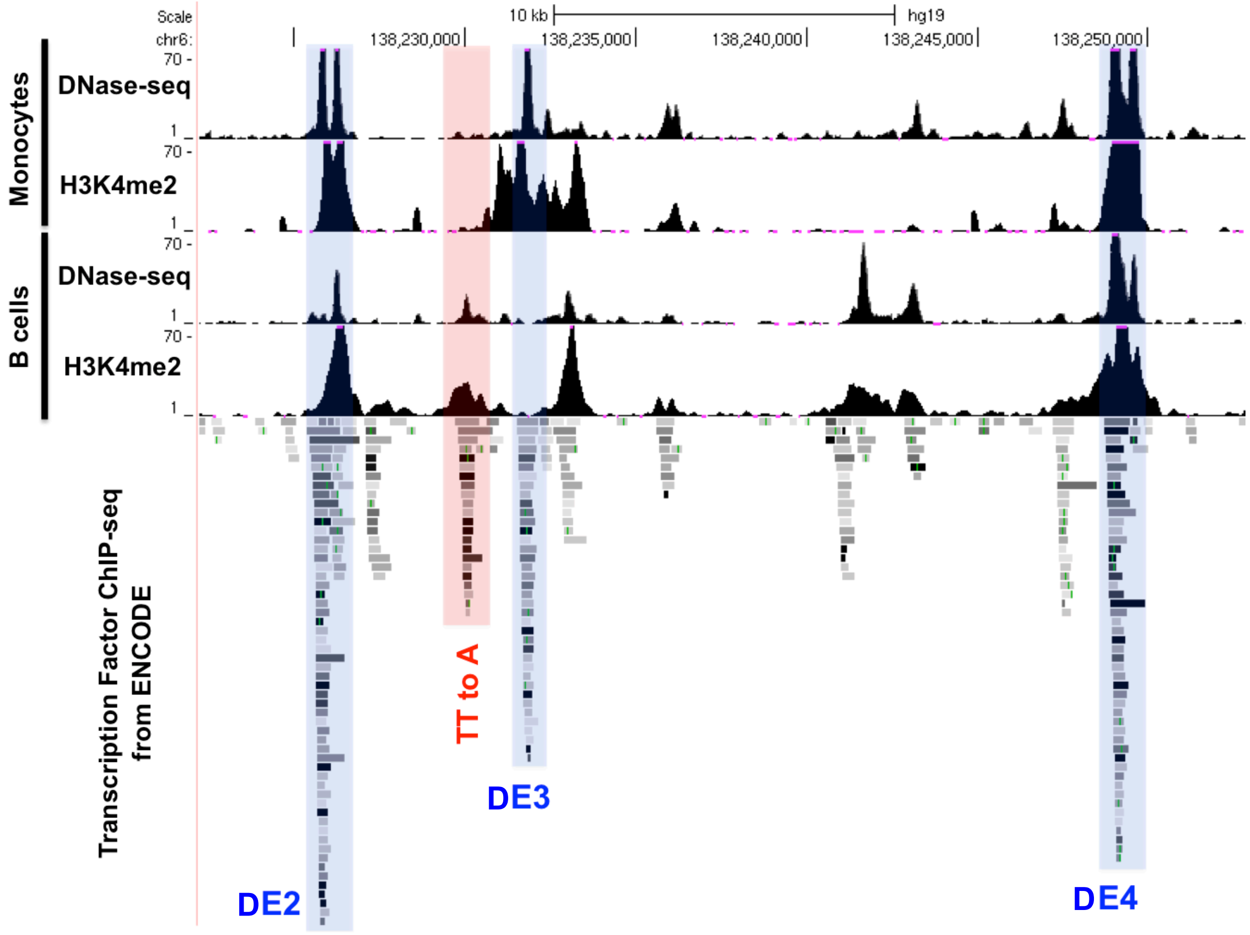

Supplementary Figure 14.

TT>A enhancer exhibits substantial TF binding despite relatively low chromatin accessibility by DNase-seq and positive histone mark H3K4me2. Gene tracks at *TNFAIP3* locus downstream of the *TNFAIP3* gene body showing downstream DE2-DE4 and TT>A enhancers. Cumulative TF binding at each enhancer in all cell types surveyed by ENCODE project is shown below the gene tracks.

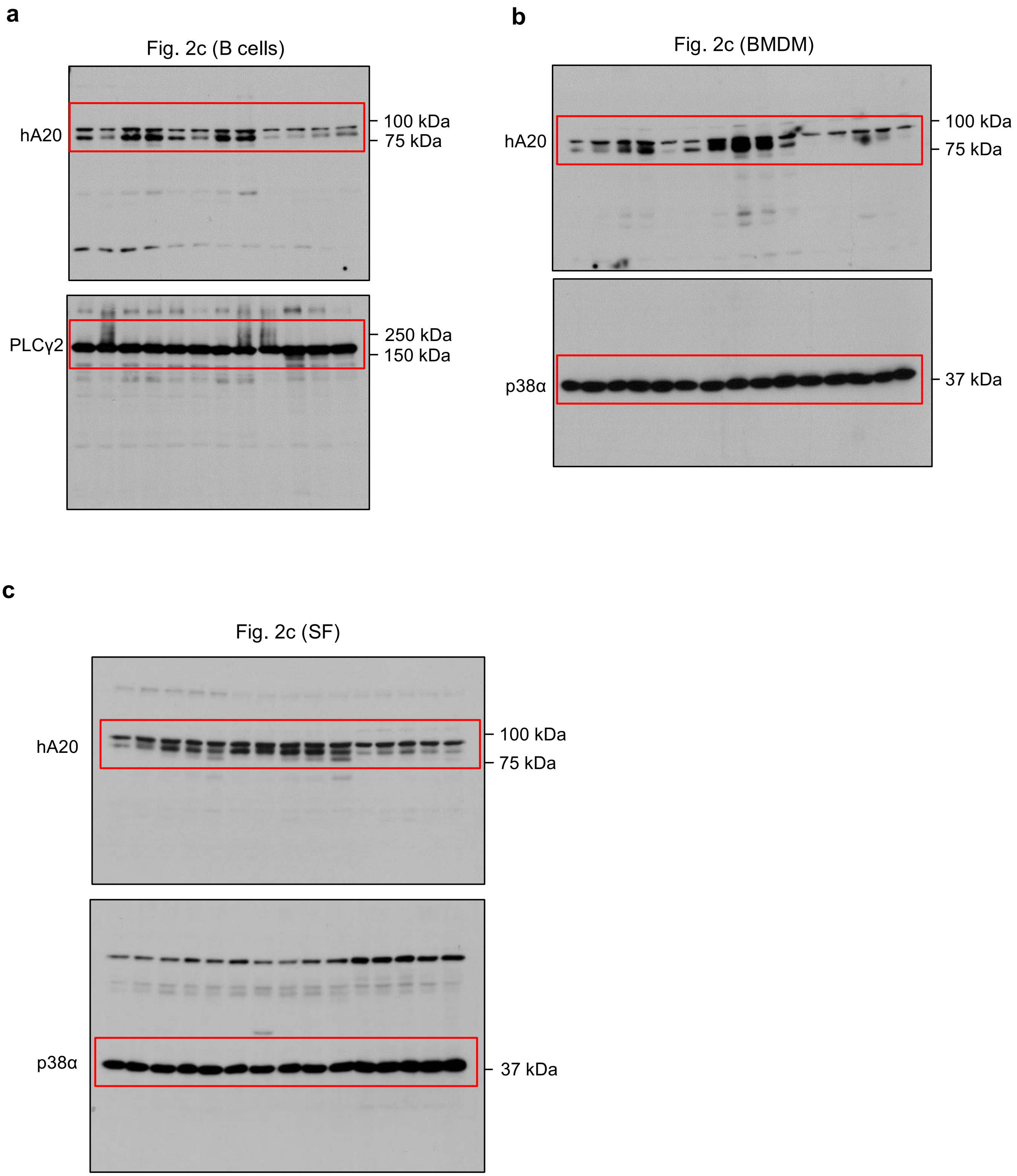

Supplementary Figure 15.

Uncropped images of the original scans of the western blots. (**a-c**) Uncropped, full-size scans of immunoblots shown in Figure 2c.

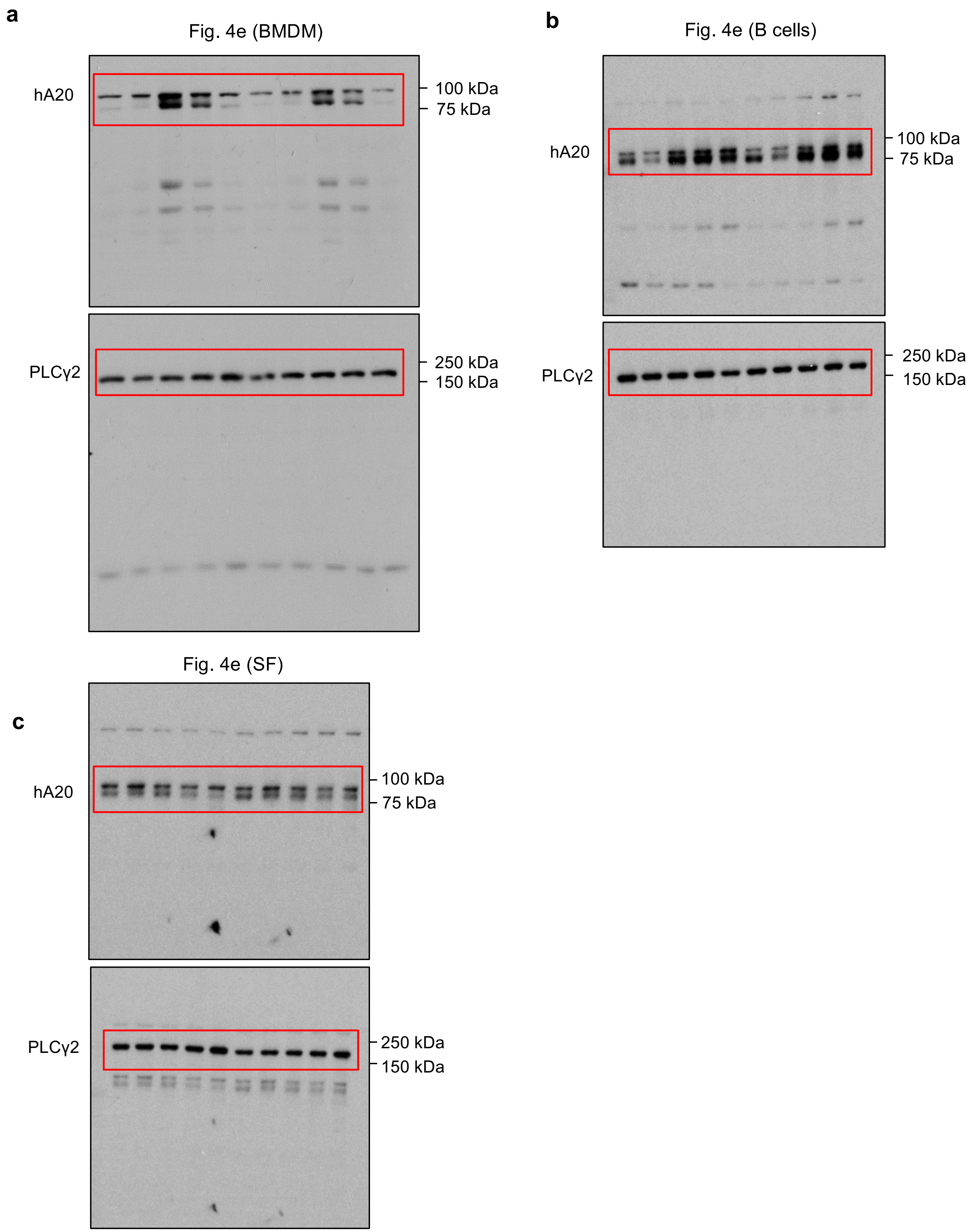

Supplementary Figure 16.

Uncropped images of the original scans of the western blots. (**a-c**) Uncropped, full-size scans of immunoblots shown in Figure 4e.

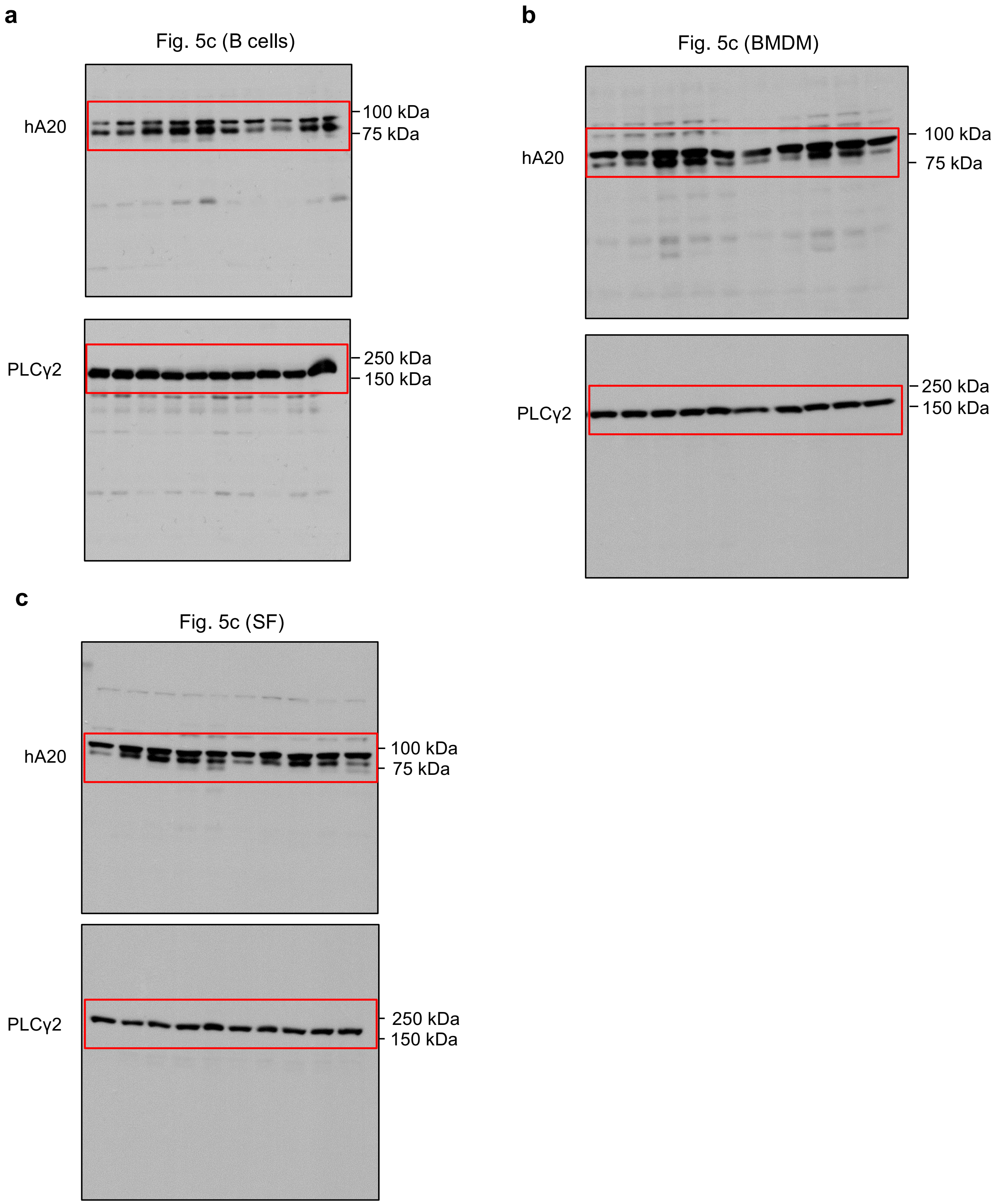

Supplementary Figure 17.

Uncropped images of the original scans of the western blots. (**a-c**) Uncropped, full-size scans of immunoblots shown in Figure 5c.

Supplementary Table 1: 5’ and 3’ ends of each BAC relative to the transcription start site of A20.

Transgenic Human BAC clones	BAC DNA length 5’ of A20 TSS	BAC DNA length 3’ of A20 TSS	Genomic coordinates
Δ-DOWN	174 kb	36.7 kb	Chr6: 138013858-138225029
CTR	76 kb	83 kb	Chr6: 138111836-138271430
Δ-UP	25.6 kb	190 kb	Chr6: 138162711-138378363

Supplementary Table 2: Mice survival at 4 weeks.

Mouse lines	Survival rate at 4 weeks
**A20-/-**	0/100
**CTR**	150/150
**∆-UP**	122/122
**∆-DOWN**	100/100

Supplementary Table 3: Human and BAC- specific primers for characterizing founder mice.

**BAC-Specific Primers to Verify Construct Integrity**		
CTR 5’ End	Forward	GCTGGGGCTCTGCTTCACCAATG
	Reverse	GGATCAAAGGGCTGGATGTGGTG
CTR 3’ End	Forward	GGAGTAGATTTGGAGGGAAGAGAG
	Reverse	GCAGACTGGTGATGTACACAGCTAGG
Δ-UP 5’ End	Forward	GAGGCTTGGGGTGTTCAGAATTGG
	Reverse	CAGCCAATGACAGCATCCCTCCTC
Δ-UP 3’ End	Forward	CTAGCAATGACCCCATGGACGCC
	Reverse	GACACTGGTCAGTCTGGAACCAT
Δ-DOWN 5’ End	Forward	CATCATCCTGAACAGCAGCCTC
	Reverse	CCTCCCACTGGAACTAAGACCTC
Δ-DOWN 3’ End	Forward	CCTTCTGTTTCCAGCAAGCATCCTCC
	Reverse	GCCTCTGCACTGGATTTTGACATCACAG

Supplementary Table 4: Primer sets for genotyping of mice.

**Primers for Colony Genotyping**		
BAC Transgene	Forward	CATAGGGAGGGAGTGATAACTC
	Reverse	GTGCTCTCCAACACCTGAAAAGG
Neomycin Cassette	Forward	CGCATGATTGAACAAGATGGATTGCACGC
	Reverse	CGCTGACAGCCGGAACACGGCGGCATC
Mouse A20	Forward	GATGTCCATATGCATCATCAG
	Reverse	CAGACATCAGAGAGCCTTGTG
DE4 deletion with CRISPR	Forward	GTCAATGCAATGGTTGTTTCAGAC
	Reverse	GAGATGGGGTTTCACCATGTTGG
TTA deletion	Forward	GATGAATGCATTGTATATTTCATTG
	Reverse	CTCCTGCTTCATGGGTCTCCTCTC

The first primer set detects the human BAC *TNFAIP3* transgene, to ensure the entire BAC is present. The second primer set detects a neomycin cassette sequence that is integrated into the mouse *Tnfaip3* locus. The third primer set encompasses the mouse *Tnfaip3* gene. These three primer sets serve to verify hA20 transgene presence and mouse A20 (mA20) absence on either allele.

Supplementary Table 5: Sequences that were deleted in Δ-DE4 and Δ-TT>A transgenic mice.

**Deleted sequences of transgenic mice**	
**DE4 with CRISPR/Cas9**	GTAAATTAAGTCAATAAACTTTTGAAGACTTGTGCCATGCTGCAGATGGACTTTAAAAGCCACTG<br>GGGGGTGGGGAGAATGGGGGTTTCTAAATATAATAAACTTCCCAAAAAGGCAATTTCAAGCCTAA<br>CTGAAGTCATACTATTTTTGAAAGTGGAAATCCCCCTTTTCACCACACAACCCCGTGGTCGAGCC<br>ATCTAAGAATTCAGCTTCTGTGCCAGTGATAGGACAAAGCTCATCCTCTGCCGAATGCCACAGTC<br>ACTCCTCTTCCTTCCTGCCACCCACAGGGTCAGCAACAAGCACTATCAGTTAAACGTGACACCTG<br>GTGTCTGCGGGCTGCCTCCGGTGGGAGCCAAATCCCACGTGTTGGTGTGGTTCCCACTTAAATA<br>GATGTTTTCCTTTGAATCGTTTCTTTTCAACCTTGATTTCAGTTCTGTGCTAGTTTCTGGACTTTTG<br>CCAGCACTTCTTCATAAAACAGCTTGTATTTGCTTTGAAAAACAAGAAAGGTTTGCATTTTGGAAA<br>ATATACTTCATACTTCATACTTCTAACCTTTACCATGGCCTGAAGGAAAAATGTAGCAGTTTCTTTC<br>TGTAAATTTTAAATATAGGAAAGATAGCGGTCACTTTATAAAGAAAGCCAAACTTGTCCACATCCT<br>TACACTCTTCCTCAAAGTTCATTTCCACATTCAATTTGGGTCGGGCTTTGTTTAGAGTGCCAATGA<br>GCCTGCCTGGGCCACTTCTGCATTCTTGATGTTTGCAGCATTCACTGCAGAAACTGGCATTAAGA<br>CAGGCCTCTGCCACCCACAGCAATGCTCCTGCCACCTCCCTCCACTGTGCAGCCCCAAAGTGAC<br>AGAGGCTGAGGATCCCCAGCAAAGCAAACTGCTTCTGTCATCTGCTTTCAGGCAATTTTGAGGC<br>ACCTTTTCTTGGCTTATTTCCACTGCTAAGCCCTGACACAATATAATATTTCAAAAGCCCCTAAGT<br>GCTGACAGAATACTCAAAATCACATTTTACAAAACCAAAGACAGAGGCCTCCCGCTGCTTATTAA<br>TGCCCATCATGATAATTCCTTACTTTGTCATAGTCTATGTATTTGTGTTGTTGTTAGTTTGTGTTGT<br>TGATTTGTTCAGCCAAGGATCAAGCAACAGAGTGCAAGTGAGGCCACCCAGCGAGGGGGAGAA<br>GGTGCAGCCTCTCAATCTACAGTGTACTTCTAAACATTCACTTTTTTTCTGATTATATAGGTAATTC<br>ACGTTTACGTGAGAAATTTTGGGGAAATTTGCAGAACATTAAGAAAAAAAAATTATTGCTCTAGAT<br>CATCTCCCCAAAATTGCACCTCAATATTTTGGGAAATATTCTTCTAGCATTTTTTTCTATGCCTTTA<br>AATATTCATCACCACCACCCCCTGCCCCTCTCCTACCCCACTCCTCCAGTTGGGATCATGTTAAA<br>CATACTGTTTCATAACAGTATGTCTAGCACACTG
**TT>A with BAC engineering**	TTTTCTCTCTTTCTCTCTGTCTCTCTCTCTCTCTCTCACACACACACACACACACACACACACACA<br>CACACCCCAGCACTCTAAACTTGAATCATTAAATGGAACCAAAAACTTCCTAAAAGTTTCACTAAA<br>ACTACCTAGGAAAACTTTGCAAAATTACCCACCCTAGAAATTGCAGTGCTAGCTCCTCCTGAGTT<br>TGTTTTGTTTTGTTTTTTTAAGTTTATTTATTTTCTGGGTACTTCTCAACAAGATGACTAGTGCATAA<br>TGCTTTATTTTCCTTTCTTTATAGGGTGGAGATAAGCTATTTCTTTTAATTTTACTATGTCTGATAG<br>AATTCCTTCATGTATGTATTGCTTTGTTTCTAAATATAAGCATGACAGGCTCAGAGCTGTCCGTGG<br>TCACGTAGGGGTTTTTGGAAATAATTTTAGCTTCGGGCATACCCCTGAACGTGGGCAGCGCCTA<br>CACATTGTCATCTCAGCCCCCTCTTACCCTTTTGGGAAGTCCTGATACCCTCATGGAGGTTTTTTT<br>TCTGCTTCATTCTCAAATCAACGTGGGGTTTCCTTATACCCTTGGCCCAACTTTAACTTACACAAT<br>TGTAACAGGCAGCATTATATTGAGAATGCATGTTGTAGAATATTGGTTCTCACCTCTGG
**TT>A with CRISPR/Cas9**	TATACTCCCACCAAGTGTGTATCTGATCCATTCTGTAATCATTTTCTCTCTTTCTCTCTGTCTCTCT<br>CTCTCTCTCTCACACACACACACACACACACACACACACACACACACCCCAGCACTCTAAACTTGAA<br>TCATTAAATGGAACCAAAAACTTCCTAAAAGTTTCACTAAAACTACCTAGGAAAACTTTGCAAAATT<br>ACCCACCCTAGAAATTGCAGTGCTAGCTCCTCCTGAGTTTGTTTTGTTTTGTTTTTTTAAGTTTATT<br>TATTTTCTGGGTACTTCTCAACAAGATGACTAGTGCATAATGCTTTATTTTCCTTTCTTTATAGGGT<br>GGAGATAAGCTATTTCTTTTAATTTTACTATGTCTGATAGAATTCCTTCATGTATGTATTGCTTTGT<br>TTCTAAATATAAGCATGACAGGCTCAGAGCTGTCCGTGGTCACGTAGGGGTTTTTGGAAATAATT<br>TTAGCTTCGGGCATACCCCTGAACGTGGGCAGCGCCTACACATTGTCATCTCAGCCCCCTCTTA<br>CCCTTTTGGGAAGTCCTGATACCCTCATGGAGGTTTTTTTTCTGCTTCATTCTCAAATCAACGTGG<br>GGTTTCCTTATACCCTTGGCCCAACTTTAACTTACACAATTGTAACAGGCAGCATTATATTGAGAA<br>TGCATGTTGTAGAATATTGGTTCTCACCTCTGGCCACAAATTTATTCACTTAGGGAATTTAAAAAA<br>AAATCCATGTCTGAGCCTTGTCCCTGAGATTCAGATTTAATTTGCCTAAGACATCACCTCTGATCG<br>TT

Supplementary Table 6: Sequences surrounding deleted regions in Δ-DE4 and Δ-TT>A transgenic mice.

**Sequence surrounding deleted regions in transgenic mice**	
**DE4 with CRISPR/Cas9**	GCAGAGCACACTGCCCAGAGTTGGGATATCAATACCCTTTGTGAAAACCACAAAATAGGCAATTT<br>GAAAAGTAGAGAGAACTCTGAAGAGGAAAAAAGGTCCCTTGCCATTTTACTACTTTCAGCATTTTA<br>ATTAAAATGTATTTTTGTCAATGCAATGGTTGTTTCAGACATTTTAAGAAAACATCTTGATTCCAAA<br>CCCCGGTGTAGTGTAATTAGTTTTCTGTCATTTAATTTGATCCATCTTTTGTTTCATTTGTTATTATA<br>AAAACCCAGAGTAATAGCCAGAGCAGATGTTATTAGCCCCATTTCACAGATTAAGAAAATGATCA<br>CACCTGTAATCTCAGCACTTTGGGAGGCCAAGGCAGGCAGATCAAGAGGTCAAGAGATCGAGAT<br>CATCCTGGCCAACATGGTGAAACCCCATCTCTATCAAAAATACAAAAATTAGCTGGGCATGGTGG<br>CACATGCCTGTAGTCCCAGCTACTCGGGAGGCTGAGACAAGAGAATTGCTTGAACTTGGGACGC<br>AGAGGTTGCAGTGACCCAAGATTGCACCACTGCGCTCCAGCCTGGCGACAGACTGTGACTCCAT<br>CTC
**TT>A with BAC engineering**	TATTATAATTAATTAGTTTTATTGCATATGTGCTGCATTCAATATTTTGCTATTATAAATAGTTATGA<br>CTTTCTATCATCCTATGATGATTCCCTCAGAATAAGTTTCTGGAATTAGAATGCAAAATTTTACATT<br>TTTTAAATAGCATTGCCAAATTATCTTCCAGAGATTTTGTTCCAATATATACTCCCACCAAGTGTGT<br>ATCTGATCCATTCTGTAATCCCATGTCTGAGCCTTGTCCCTGAGATTCAGATTTAATTTGCCTAAG<br>ACATCACCTCTGATCGTGGATGGGTGTCTTAATGTGGATTCCATGGTCTACCACTCTAGATGATC<br>ACAATGAGCTCTCCAAAGAAAAATGAGGCTGGATAAAGACAGGAACTGAGCACAAGAGAGGAGA<br>CCCATGAAGCAGGAGTAAAAGAAAATCTCTCTCCTTTTGTGGTTTTAAAGTAGAAGAAGGTTAAAT<br>CGTGTCAGTTTATGAAAAGTAGAGGATGTGAGAATTTCATCATTTTATCACCATCTTTGGTATCTC<br>CAATTCTGCTCAGTAATCTGCTTAACAAATCATGAGTATTTTCAATAGAAGGTATAAGTTCAT
**TT>A with CRISPR/Cas9**	TATTATAATTAATTAGTTTTATTGCATATGTGCTGCATTCAATATTTTGCTATTATAAATAGTTATGA<br>CTTTCTATCATCCTATGATGATTCCCTCAGAATAAGTTTCTGGAATTAGAATGCAAAATTTTACATT<br>TTTTAAATAGCATTGCCAAATTATCTTCCAGAGATTCTGGATAAAGACAGGAACTGAGCACAAGA<br>GAGGAGACCCATGAAGCAGGAGTAAAAGAAAATCTCTCTCCTTTTGTGGTTTTAAAGTAGAAGAA<br>GGTTAAATCGTGTCAGTTTATGAAAAGTAGAGGATGTGAGAATTTCATCATTTTATCACCATCTTT<br>GGTATCTCCAATTCTGCTCAGTAATCTGCTTAACAAATCATGAGTATTTTCAATAGAAGGTATAAG<br>TTCAT

Supplementary Table 7: List of primer sets utilized for qPCR in FAIRE assays.

Faire primers	Sequence	
	Forward primer	Reverse primer
A20 promoter	CTCTGGCGGCCGGCTGGACGCAC	GGCTCCAAGCTCGCTTGGCCCG
DE2	TCTGCATCTCCTCAACCACAC	AAACTCGGTAACCAGCCTCC
DE3	GACTGTTCTGCTTGCCTCCT	GACCTCAGCCAAAACTGGGA
DE4	CCGTGGTCGAGCCATCTAAG	AGGAGTGACTGTGGCATTCG
eTTA	GTGGTCACGTAGGGGTTTTTG	GAGGGGGCTGAGATGACAAT
UE1	CCCTTCAGCTTCTGCTCTACC	CAGACTTAAGGCCCGACGAT
UE2	GCATGACGCACAGAAGAGTC	GAGACCCAAAGGGATCAGGC
UE3	GACACAGCCCTACAAATTGCC	GGTCAGCTAACAGAGCAGGA
cntrl	ACCATTGCCCATGCATCAGA	AGAAAGATCCCACTGGCTGC

The panel below shows the genomic location of the primers used relative to DNase-seq tracks of CD14+ monocytes and B-cells from ENCODE.

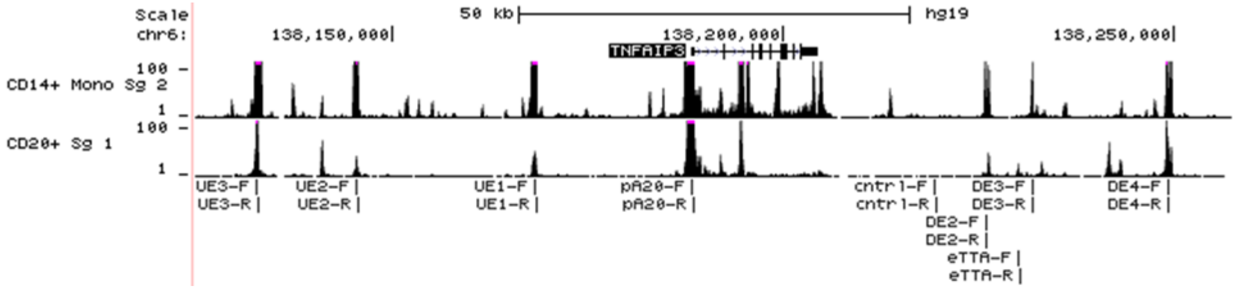

Supplement: Supplementary file 1 — Supplementary Information [file 41467_2018_3081_MOESM1_ESM.pdf]
